# Supplementary material for: Donor genetic variants in interleukin-6 and interleukin-6 receptor associate with biopsy-proven rejection following kidney transplantation
Source: Sci Rep. 2021 Aug 13;11:16483. doi: 10.1038/s41598-021-95714-z (PMC8363661; doi:10.1038/s41598-021-95714-z)
Supplement: Supplementary file 1 — Supplementary Information. [file 41598_2021_95714_MOESM1_ESM.docx]

**Supplementary data**

**Supplementary Figure S1:**

**Kaplan-Meier curves for rejection-free survival of kidney allografts transplanted in the 1990s and in the 2000s according to the interleukin-6 receptor polymorphism.**

**
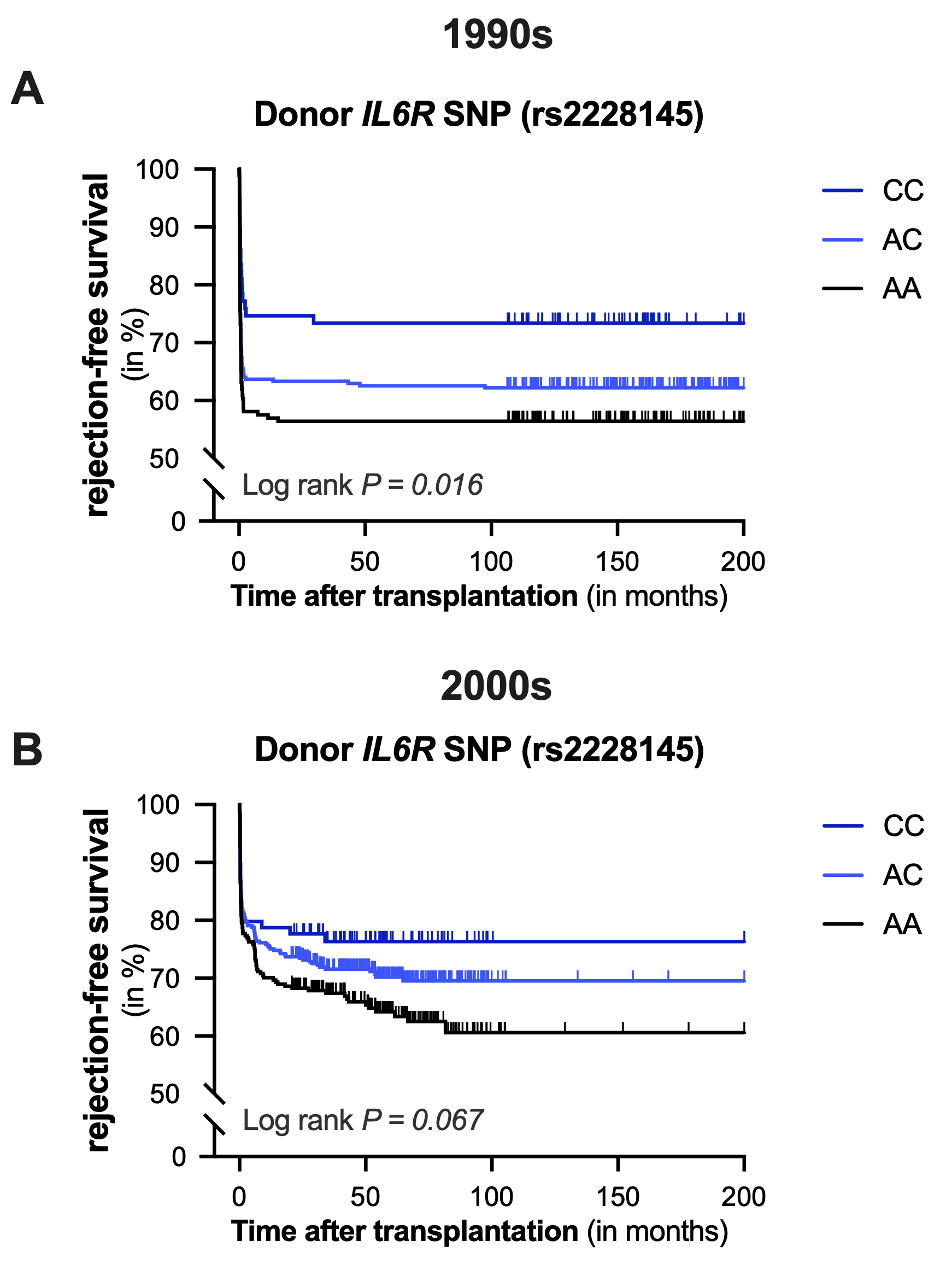
**

Cumulative rejection-free survival of renal allografts according to the presence of the IL-6 receptor polymorphism *(IL6R, rs2228145 A>C, previously rs8192284)* in the donor for patients transplanted in the (A) 1990s and in the (B) 2000s. Log-rank test was used to compare the incidence of biopsy-proven rejection between the groups.

**Supplementary Figure S2: Kaplan-Meier curves for rejection-free survival of kidney allografts according to donor-recipient mismatches for the interleukin-6 and interleukin-6 receptor polymorphism.**

**
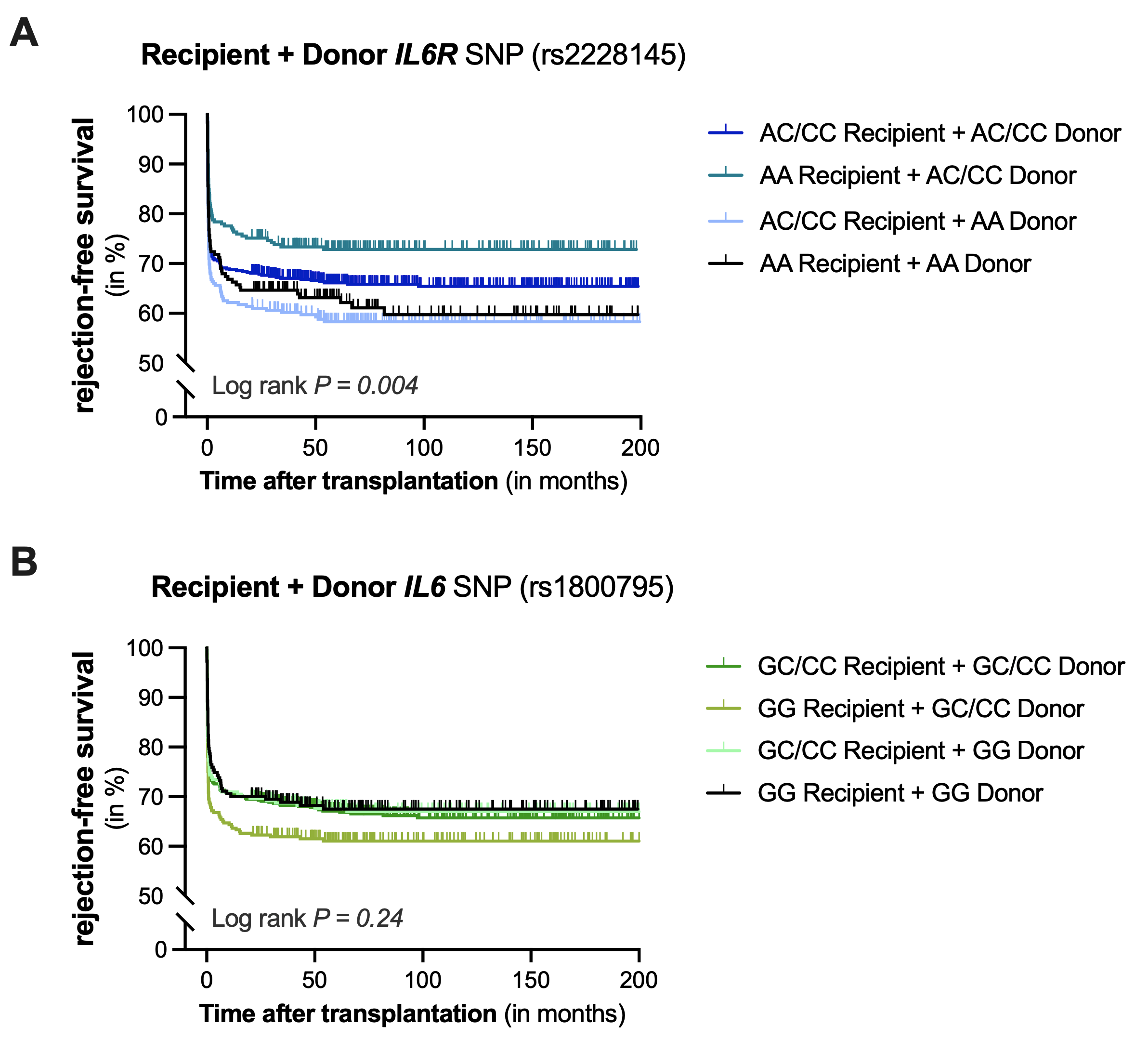
**

To assess whether donor-recipient mismatches for common polymorphisms increase the risk for biopsy-proven rejection, kidney transplant pairs were divided into four groups according to the presence of the minor allele in the donor and recipient. Cumulative rejection-free survival of renal allografts according to the presence of (A) the IL-6 receptor polymorphism *(IL6R, rs2228145 A>C, previously rs8192284)* and (B) the interleukin-6 polymorphism (*IL6, rs1800795 G>C)* in donor-recipient pairs. Log-rank test was used to compare the incidence of biopsy-proven rejection between the groups.
